# Waiting time for cancer treatment and mental health among patients with newly diagnosed esophageal or gastric cancer: a nationwide cohort study

**DOI:** 10.1186/s12885-016-3013-7

**Published:** 2017-01-03

**Authors:** Huan Song, Fang Fang, Unnur Valdimarsdóttir, Donghao Lu, Therese M.-L. Andersson, Christina Hultman, Weimin Ye, Lars Lundell, Jan Johansson, Magnus Nilsson, Mats Lindblad

**Affiliations:** 1 Department of Medical Epidemiology and Biostatistics, Karolinska Institutet, Box 281, Stockholm, SE171 77 Sweden; 2 Center of Public Health Sciences, Faculty of Medicine, University of Iceland, Reykjavík, Iceland; 3 Department of Epidemiology, Harvard T.H. Chan School of Public Health, Boston, MA USA; 4 Department of Documentation & Quality, Danish Cancer Society, Copenhagen, Denmark; 5 Division of Surgery, Department of Clinical Science Intervention and Technology, Karolinska Institutet and Centre for Digestive Diseases, Karolinska University Hospital, Stockholm, Sweden; 6 Department of Surgery, Skåne University Hospital, Lund, Sweden

**Keywords:** Mental disorder, Gastric cancer, Esophageal cancer, Cohort study, Cancer treatment, Waiting time

## Abstract

**Background:**

Except for overall survival, whether or not waiting time for treatment could influences other domains of cancer patients’ overall well-being is to a large extent unknown. Therefore, we performed this study to determine the effect of waiting time for cancer treatment on the mental health of patients with esophageal or gastric cancer.

**Methods:**

Based on the Swedish National Quality Register for Esophageal and Gastric Cancers (NREV), we followed 7,080 patients diagnosed 2006–2012 from the time of treatment decision. Waiting time for treatment was defined as the interval between diagnosis and treatment decision, and was classified into quartiles. Mental disorders were identified by either clinical diagnosis through hospital visit or prescription of psychiatric medications. For patients without any mental disorder before treatment, the association between waiting time and subsequent onset of mental disorders was assessed by hazard ratios (HRs) with 95% confidence interval (CI), derived from multivariable-adjusted Cox model. For patients with a preexisting mental disorder, we compared the rate of psychiatric care by different waiting times, allowing for repeated events.

**Results:**

Among 4,120 patients without any preexisting mental disorder, lower risk of new onset mental disorders was noted for patients with longer waiting times, i.e. 18–29 days (HR 0.86; 95% CI 0.74-1.00) and 30–60 days (HR 0.79; 95% CI 0.67-0.93) as compared with 9–17 days. Among 2,312 patients with preexisting mental disorders, longer waiting time was associated with more frequent psychiatric hospital care during the first year after treatment (37.5% higher rate per quartile increase in waiting time; *p for trend* = 0.0002). However, no such association was observed beyond one year nor for the prescription of psychiatric medications.

**Conclusions:**

These data suggest that waiting time to treatment for esophageal or gastric cancer may have different mental health consequences for patients depending on their past psychiatric vulnerabilities. Our study sheds further light on the complexity of waiting time management, and calls for a comprehensive strategy that takes into account different domains of patient well-being in addition to the overall survival.

**Electronic supplementary material:**

The online version of this article (doi:10.1186/s12885-016-3013-7) contains supplementary material, which is available to authorized users.

## Background

Given that the impact of tumor stage on survival is undisputed, it is problematic that the majority of tumors are diagnosed at a relatively advanced stage, when accelerated tumor growth and metastasis formation are imminent [1, 2]. Therefore, it is logical to believe that a timely diagnostic work-up and treatment are of critical importance for the subsequent disease course and survival of the patients. However, previous studies, either from observational investigations [3, 4] or interventional studies (‘Cancer waiting times targets’ project [5], introduced by UK government in 2000 [6]) failed to show a clear survival benefit of shortened waiting time for cancer treatment. This was true even for relatively aggressive cancers, e.g. esophageal, gastric cancers [7, 8] and pancreatic cancer [6]. Yet, whether or not shorter waiting time for treatment influences other domains of cancer patients’ overall well-being is to a large extent unknown.

Cancer patients have been shown to have increased risks of multiple mental disorders both immediately after cancer diagnosis and later on [9, 10]. Knowledge about determinants for such risk increase may shed light on potential interventions. Due to the multimodality cancer diagnostics and therapies to date, waiting time for cancer treatment is likely to be increasing [11–13]. There is therefore an urgent need to clarify the impact of the extended waiting time on patient well-being in general, including the subsequent risk of mental disorders. To this end, we performed a cohort study to address the effect of waiting time for cancer treatment on the mental health of patients with newly diagnosed esophageal or gastric cancer.

## Methods

### Database and study design

Our study was based on the National Registry for Esophageal and Gastric Cancers (NREV), which included all patients with a diagnosis of esophageal or gastric cancer in Sweden. Details about this register, including its validity, have been described elsewhere [14]. Briefly, the register was officially launched in 2006, where patients were recruited from all health care providers diagnosing and managing gastric and esophageal cancers in Sweden. With regular checking with the Swedish Cancer Register to identify any potential additional patients, the NREV database has an average coverage of 92% for each calendar year [14]. Comprehensive information regarding diagnosis and treatment (mainly operations) was collected through questionnaires completed by the responsible physicians. The register was further cross-linked to the nationwide Cause of Death Register, Patient Register, Prescribed Drug register, and Emigration Register, obtaining information on follow-up outcomes of these patients.

In total, 7,984 patients with either an esophageal or gastric cancer were registered in NREV during 2006–2012. We excluded patients with conflict information (died or emigrated before diagnosis, *n* = 60), or with missing (567) or incorrect (275) information on the date of treatment decision, leaving 7,080 patients (88.7%) in the present analyses. All patients were followed until death, emigration out of Sweden, or December 31, 2012, whichever occurred first. This study was approved by the Regional Ethical Review Board in Stockholm, Sweden (Dnr 2013/596-31/3). Since we used de-identified register data, individual informed consent was not sought in line with institutional regulations.

### Waiting time for cancer treatment

Since the exact starting date of cancer treatment was not available for patients without surgical treatment in NREV, we used the date of treatment decision as a proxy of treatment initiation for all participants. Waiting time for cancer treatment was accordingly defined as the time interval between cancer diagnosis and treatment decision. For most of the patients, treatment started several days after treatment decision. For patients that received primary surgical treatment (22%), however, the actual waiting time until surgery might be postponed because of suboptimal physical status, routine pre-operation exams, or simply a queue to surgery. Nevertheless, a strong correlation was detected between the waiting time from cancer diagnosis to surgery and the waiting time between diagnosis and treatment decision (Pearson correlation coefficient = 0.86).

### Ascertainment of mental disorders

We ascertained mental disorders in two ways. Through cross-linkage to the Swedish Patient Register, we identified all inpatient or outpatient hospital visits with a mental disorder as one of the discharge or outpatient diagnoses, using the 10th Swedish revision of International Classification of Diseases codes (ICD-10: F10-F99). To complement the definition of mental disorders by using hospital diagnoses alone, we additionally assessed the use of psychiatric medications, by linking the cohort to Prescribed Drug Register. The selected psychiatric medications included antipsychotics (ATC code: N05A), anxiolytics (N05B) and antidepressants (N06A). Patients with any hospital visit or drug prescription with the above-mentioned ICD or ATC codes before treatment decision were defined as having a preexisting mental disorder. In sub-analyses, we specifically examined depression (ICD10: F32 or F33; ATC code N06A) and anxiety (ICD10: F40 or F41; ATC code N05B).

### Statistical analyses

We first presented the basic characteristics of patients with different waiting times for treatment. The patients with a waiting time greater than 60 days (*n* = 648, 9.2%) were then excluded from the following analyses, since such long delay was unusual and might allegedly reflect additional complexity of the disease or a strong wish of the patients. The remaining cancer patients were classified into four groups (≤8 days, 9–17 days, 18–29 days, or 30–60 days), according to quartile distributions. Because a large proportion of patients presented with a history of mental disorder before treatment decision (35%), we performed separately the primary analyses for patients with and without such history.

#### Patients without previous mental disorders

For 4,120 patients without any mental disorders before treatment decision, we followed them from date of treatment decision until first diagnosis of mental disorder (captured by either hospital visit or prescription of psychiatric drugs), emigration, death, or December 31, 2012, whichever occurred first. The association between waiting time and risk of mental disorder was examined by comparing the respective waiting time groups. We calculated hazard ratios (HRs) with their 95% confidence intervals (CIs) by Cox model, adjusting for age at diagnosis, sex, marital status (single, married, divorce, or widow/widower), education level (<9 years, 9–12 years, or >12 years), physical status (The American Society of Anesthesiologists (ASA) classification <2, or 2 and above), cancer type (esophageal or gastric cancer), pathological/clinical (if pathological stage unavailable) stage (0-II stage, III stage, or IV stage), planned treatment type (curative, palliative, or supportive treatment), multidisciplinary (MDC) meeting (yes or no), admission pathway (general physician referral or emergency visit), and hospital volume (low, median, or high). Since a dramatically increased risk of mental disorders around cancer diagnosis has been reported [10], we used time since cancer diagnosis as the underlying timescale for Cox models after taking into account the delayed cohort entry (the number of days between cancer diagnosis and treatment decision). The proportional hazards assumption was checked graphically by Schoenfeld’s partial residuals. Neither waiting time groups nor the above covariates appeared to violate this assumption. Cumulative incidence curves of mental disorders for different waiting time groups were graphed using the Nelson-Aalen method.

To assess potential effect modifiers of the studied associations, we performed sub-group analyses by different characteristics (e.g. sex, age group, etc.). We then separately analyzed mental disorders identified through hospital diagnosis alone and psychiatric medications alone assuming that mental disorders identified through hospital visits represent a severer phenotype compared to mental disorders identified through prescribed medications alone. Lastly, in addition to lumping together all mental disorders, we individually assessed HRs for depression and anxiety disorders.

#### Patient with previous mental disorders

For 2,179 patients with a mental disorder history before cancer diagnosis and 133 patients with a newly onset mental disorder during the waiting time for treatment, we compared the rate of psychiatric care (either through hospital visit or prescription of psychiatric medication) after treatment decision between different waiting time groups by extended Cox models (counting process model) [15], enabling inclusion of repeated events. Due to the overall violated proportional hazard assumption we divided the follow-up period (<1 year and ≥1 year) and fitted separate models. The Cox models were adjusted for all covariates stated above.

#### Sensitivity analysis

A sensitivity analysis was conducted among patients that received surgical treatment alone (without preoperative treatment, *n* = 1618), for whom the waiting time to the actual treatment (surgery) could be estimated precisely. We repeated all the above-mentioned analyses to validate the results of the main analyses.

To assess potential impact of different time scales on the study results, we further used time since treatment decision, as well as time since 60 days after cancer diagnosis, as the underlying time scales in the Cox models.

A *p* value less than 0.05 was considered to be statistically significant. All analyses were conducted in SAS statistical software, version 9.4 (Cary, NC).

## Results

Table 1 illustrates the baseline characteristics of all participants, as well as by the different groups of waiting time for treatment. Overall, the mean age at the time of cancer diagnosis was 74 years and 65.7% of the patients were male. Patients with shorter waiting times generally had more advanced disease. In particular, patients with the shortest waiting time (1–8 days) tended to be older, with worse physical status, more advanced cancer stage and were more likely to be admitted through emergency visit. Most of these patients received their treatment decision without an MDC meeting (60.8%). Since patients with the shortest waiting time obviously differed from the other patients, we used the second shortest waiting time group (‘9-17 days’) as the reference group in all following analyses.

**Table 1:** Characteristics of patients with a newly diagnosed esophageal or gastric cancer, by different waiting time groups

Characteristics	All	Waiting time groups				
		1–8 days	9–17 days	18–29 days	30–60 days	>60 days
Number of patients	7080	1733	1542	1556	1601	648
Demographic factors						
Age, mean ± SD, years	71.4 ± 11.9	73.7 ± 12.5	70.4 ± 12.2	70.4 ± 11.6	71.1 ± 11.1	70.3 ± 11.1
Gender (% male)	65.7	63.1	67.1	66.3	65.5	69.0
Material status, n (%)						
Single	914 (12.9)	235 (13.6)	227 (14.7)	180 (11.6)	189 (11.8)	83 (12.8)
Married	3626 (51.2)	797 (46.0)	806 (52.3)	843 (54.2)	842 (52.6)	338 (52.2)
Divorce	1158 (16.4)	284 (16.4)	236 (15.3)	242 (15.6)	284 (17.7)	112 (17.3)
Widow/widower	1368 (19.3)	411 (23.7)	271 (17.6)	288 (18.5)	283 (17.7)	115 (17.8)
Missing	14 (0.2)	6 (0.3)	2 (0.1)	3 (0.2)	3 (0.2)	0 (0.0)
Education level, n (%)						
< =9 years	3070 (43.4)	778 (44.9)	655 (42.5)	643 (41.3)	700 (43.7)	294 (45.4)
10–12 years	2561 (36.2)	586 (33.8)	545 (35.3)	578 (37.2)	600 (37.5)	252 (38.9)
> =12 years	1038 (14.7)	227 (13.1)	239 (15.5)	255 (16.4)	235 (14.7)	82 (12.7)
Missing	411 (5.8)	142 (8.2)	103 (6.7)	80 (5.1)	66 (4.1)	20 (3.1)
Disease-related factors						
Time of follow-up (months, until death, emigration or the end of study)						
Mean ± SD	15.6 ± 18.0	10.4 ± 16.1	13.0 ± 15.8	16.3 ± 17.7	18.5 ± 18.0	26.6 ± 21.5
Hospital Volume						
Low (<20 cases/year)	3310 (46.8)	975 (56.3)	749 (48.6)	664 (42.7)	659 (41.2)	263 (40.5)
Median (20–40 cases/year)	1868 (26.4)	442 (25.5)	456 (29.5)	420 (27.0)	387 (24.1)	163 (25.2)
High (>40 cases/year)	1899 (26.8)	314 (18.1)	337 (21.9)	471 (30.3)	555 (34.7)	222 (34.3)
Missing	2 (0.0)	0 (0.0)	1 (0.0)	0 (0.0)	0 (0.0)	3 (0.0)
ASA physical status, n (%)						
I-II	4319 (61.0)	902 (52.1)	970 (62.9)	1023 (65.8)	1030 (64.3)	394 (60.7)
III-IV	2588 (36.6)	791 (45.6)	529 (34.3)	494 (31.8)	539 (33.7)	235 (36.3)
Missing	173 (2.4)	40 (2.3)	43 (2.8)	39 (2.4)	32 (2.0)	19 (2.9)
Cancer type, n (%)						
Gastric cancer	3562 (50.3)	1004 (57.9)	788 (51.1)	737 (47.4)	715 (44.7)	318 (49.1)
Esophageal cancer	3518 (49.7)	729 (42.1)	754 (48.9)	819 (52.6)	886 (55.3)	330 (50.9)
Cancer stage, n (%)						
Stage 0	191 (2.70)	24 (1.38)	24 (1.56)	29 (1.86)	52 (3.25)	62 (9.57)
Stage I	905 (12.8)	131 (7.56)	157 (10.2)	226 (14.5)	260 (16.2)	131 (20.2)
Stage II	924 (13.1)	155 (8.94)	200 (13.0)	252 (16.2)	236 (14.7)	81 (12.5)
Stage III	2824 (39.9)	688 (39.7)	601 (39.0)	615 (39.5)	671 (41.9)	249 (38.4)
Stage IV	2188 (30.9)	726 (41.9)	557 (36.1)	423 (27.2)	369 (23.1)	113 (17.4)
Missing	48 (0.7)	9 (0.5)	3 (0.2)	11 (0.7)	13 (0.8)	12 (1.9)
Admission type, n (%)						
GP referral	5406 (76.4)	1071 (61.8)	1128 (73.2)	1303 (83.7)	1367 (85.4)	537 (82.9)
Emergency intake	1517 (21.4)	627 (36.2)	367 (23.8)	225 (14.5)	201 (12.6)	97 (15.0)
Missing	157 (2.2)	35 (2.0)	47 (3.0)	28 (1.8)	33 (2.0)	14 (2.1)
Treatment decided through multidisciplinary conference, n (%)						
Yes	4080 (57.6)	668 (38.6)	929 (60.3)	990 (63.6)	1074 (67.1)	419 (64.7)
No	2953 (41.7)	1053 (60.8)	599 (38.9)	556 (35.7)	521 (32.5)	224 (34.6)
Missing	47 (0.7)	12 (0.6)	14 (0.8)	10 (0.7)	6 (0.4)	5 (0.7)
Planned treatment type, n (%)						
Curative	3038 (42.9)	484 (27.9)	607 (39.4)	783 (50.3)	824 (51.5)	340 (52.5)
Palliative	2631 (37.2)	714 (41.2)	663 (43.0)	547 (35.2)	548 (34.2)	159 (24.5)
Supportive	1400 (19.8)	531 (30.6)	271 (17.6)	226 (14.5)	227 (14.2)	145 (22.4)
Missing	11 (0.1)	4 (0.3)	1 (0.0)	0 (0.0)	2 (0.1)	4 (0.6)

*SD* standard deviation, *ASA* American Society of Anesthesiologists

### Patients without previous mental disorders

Among patients without a previous history of mental disorder, 1,268 developed a mental disorder during follow-up. In general, we observed a decreased risk of mental disorder for patients with longer waiting times, compared to the reference group (‘9–17 days’) (Table 2); the multivariable adjusted HRs for all mental disorders were 1.07 (95% CI 0.91–1.25) for a waiting time of ‘1–8 days’, 0.86 (95% CI 0.73–1.01) for ‘18–29 days’, and 0.79 (95% CI 0.67–0.93) for ‘30–60 days’. A clear trend of decreasing HRs with increasing waiting time was also noted (*p* for trend < 0.0001). Similar patterns of HRs were noted for mental disorders identified through hospital diagnosis or through drug prescription alone. The results for depression and anxiety did not differ largely from all mental disorders, although patients with the shortest waiting time appeared to have higher risk of anxiety, but not depression, compared to the reference group (Table 2).

**Table 2:** Hazard ratios (HRs) and 95% confidence intervals (CIs)^*^ for mental disorders among patients without mental disorder history (*n* = 4,120), by different waiting time groups

Outcomes	Number of cases	1–8 days	9–17 days	18–29 days	30–60 days
All mental disorders	1268	1.07 (0.91–1.25)	1.00 (reference)	0.86 (0.73–1.01)	0.79 (0.67–0.93)
Mental disorders identified by in-/out-patient diagnosis	129	0.79 (0.47–1.34)	1.00 (reference)	0.85 (0.54–1.33)	0.56 (0.34–0.93)
Mental disorders identified through drug prescription	1227	1.09 (0.93–1.29)	1.00 (reference)	0.88 (0.75–1.04)	0.82 (0.69–0.97)
Depression (ICD10:F32,F33 or/and ATC N06A)	317	0.96 (0.69–1.34)	1.00 (reference)	0.91 (0.67–1.23)	0.66 (0.48–0.92)
Anxiety (ICD10:F40,F41 or/and ATC N05B)	799	1.21 (0.99–1.48)	1.00 (reference)	0.87 (0.71–1.06)	0.84 (0.68–1.03)

*Adjusted for age, sex, marital status (single, married, divorce, widow/widower), education level (<9 years, 9–12 years, >12 years), physical status (The American Society of Anesthesiologists (ASA) classification <2, 2 and above), cancer type (esophageal/gastric cancer), stage (0, I, II, III, IV stage), and planned treatment type (curative, palliative, or supportive treatment), multidisciplinary meeting (yes/no), admission pathway (general physician referral / emergency intake), hospital volume (low, median, high)

**Fig. 1:**
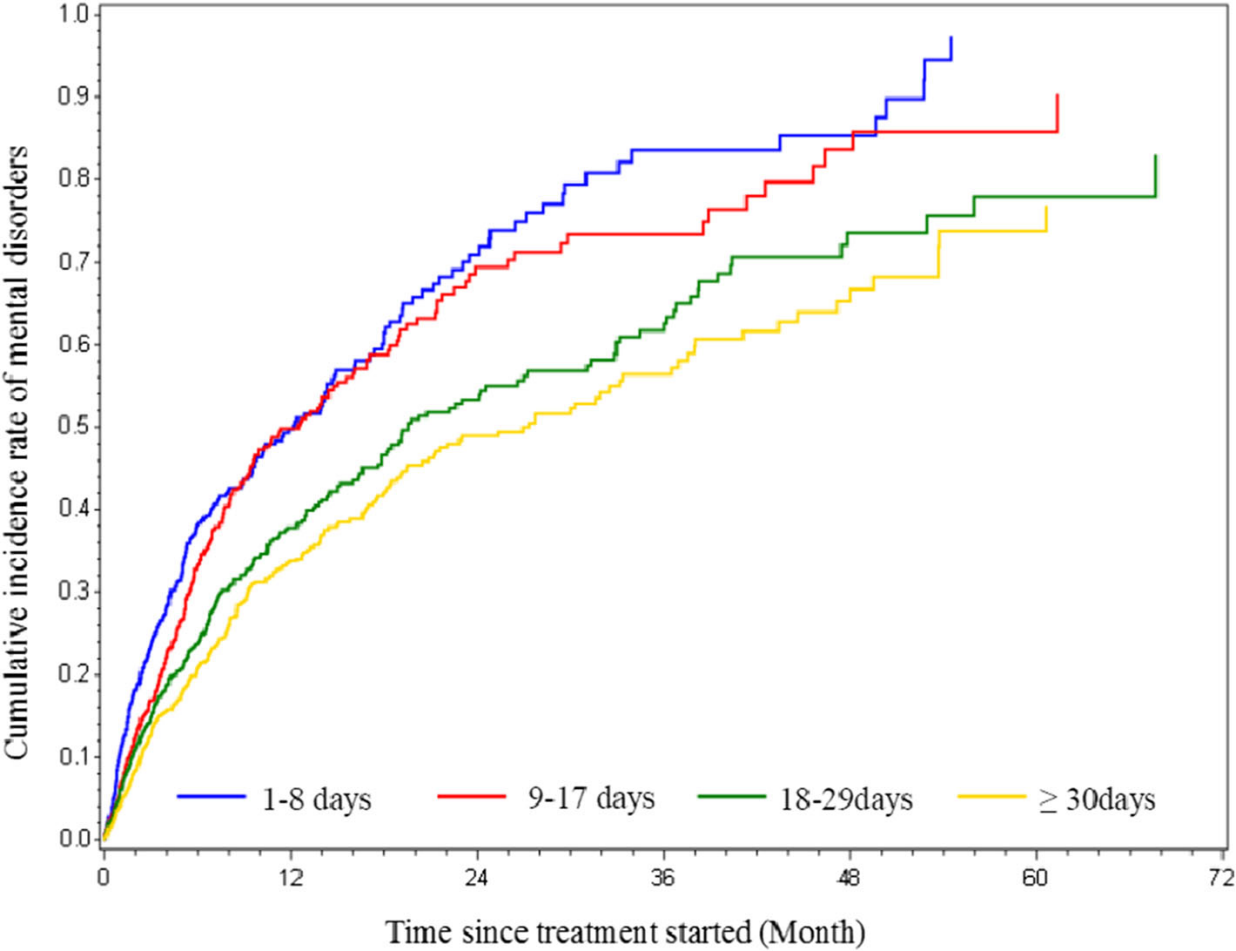
Cumulative incidence rates of mental disorders among patients without preexisting mental disorders

Time since treatment decision (follow-up period), psychical status, cancer type, cancer stage, age group, education level, marital status, whether or not having MDC meeting, treatment plan or hospital volume did not modify these results further (Additional file 1: Table S1). The cumulative incidence curves (Fig. 1) illustrate the occurrence of mental disorders during follow-up by different groups of waiting times (since the curves didn’t adjust for any covariates, they actually reflect a hypothetical scenario that all patients would survive during follow-up) and show that patients with longer waiting times had lower cumulative incidence rates of mental disorders after treatment decision.

### Patients with previous mental disorders

Among 2179 patients with previous history of mental disorders, 68.8% received psychological care after the decision date of cancer treatment. Prolonged waiting time for cancer treatment was associated with an increased rate of hospital visits for mental disorders during the first year after treatment (Table 3), illustrating a 37.5% higher rate of psychiatric hospital care per increasing waiting time (95% CI 15.6–63.5%, *p* for trend = 0.0002). This risk elevation was more pronounced for patients with only a history of psychiatric medication use but no hospital visit for mental disorders before treatment (*n* = 1,361), for whom the rate of hospital visits increased by 49.0% per increasing waiting time group (95% CI 18.4%–87.4%) during the first year after treatment. The pattern was less clear beyond one year after treatment decision or for psychiatric medication use (Table 3).

**Table 3:** Comparison of the rate of psychiatric care among patients with preexisting mental disorders (*n* = 2,312), by different waiting time groups

Waiting time group	Hazard Ratios (HRs) and 95% Confidence Intervals (CIs)^a^					
	All psychiatric cares		Hopsital visits		Medication prescriptions	
	≤1 year	>1 year	≤1 year	>1 year	≤1 year	>1 year
1–8 days	0.90 (0.74–1.10)	0.68 (0.44–1.07)	0.80 (0.49–1.31)	0.36 (0.14–0.91)	0.90 (0.74–1.10)	0.72 (0.46–1.13)
9–17 days	1.00 (reference)	1.00 (reference)	1.00 (reference)	1.00 (reference)	1.00 (reference)	1.00 (reference)
18–29 days	1.04 (0.86–1.26)	0.84 (0.51–1.24)	1.49 (0.98–2.25)	0.80 (0.38–1.72)	1.01 (0.83–1.23)	0.79 (0.51–1.22)
30–60 days	1.09 (0.91–1.32)	0.70 (0.47–1.02)	2.01 (1.24–3.24)	0.51 (0.24–1.07)	1.03 (0.85–1.24)	0.71 (0.48–1.04)

^a^Estimated by extended Cox model, adjusting for age, sex, marital status (single, married, divorce, widow/widower), education level (<9 years, 9–12 years, >12 years), physical status (The American Society of Anesthesiologists (ASA) classification <2, 2 and above), cancer type (esophageal/gastric cancer), stage (0, I, II, III, IV stage), and planned treatment type (curative, palliative, or supportive treatment), multidisciplinary meeting (yes/no), admission pathway (general physician referral / emergency intake), hospital volume (low, median, high)

### Sensitivity analysis

Analyses restricted to patients that received surgical treatment alone yielded similar results as the primary analyses, with the exception that among patients with a history of psychiatric disorders (*n* = 473), the elevated rate of hospital visit for mental disorders by longer waiting times appeared also beyond one year after treatment (Additional file 2: Table S2). Alternative underlying time scales barely changed the results (data not shown).

## Discussion

The impact of waiting time for treatment has mostly been studied in terms of cancer survival among patients with esophageal or gastric cancer [8, 16]. To the best of our knowledge, this is the first large nationwide cohort study describing the association between waiting time for cancer treatment and mental health in patients with esophageal or gastric cancer. Our study sheds further light on the complexity of waiting time management, by highlighting a need to take into account different domains of patient well-being in addition to the overall survival. The complexity is further reflected by the potential differential impact of waiting time for treatment for different patients, depending on past psychiatric vulnerabilities. Specifically, while patients without any previous mental disorders may benefit from longer waiting times, in terms of future mental health, patients with previous mental disorders seem to benefit from a quick treatment decision.

Long waiting times for diagnosis and treatment are always of concern for the patients and the healthcare providers [5]. In Sweden, factors that could affect the waiting time of cancer treatment decision include the speed of the completion of staging information (mainly related to waiting time for radiological examinations), patients’ wishes, as well as availability of MDC meeting (which normally hold once per week, meaning up to six days waiting time for some patients). According to our NREV database, 70% of the patients with esophageal or gastric cancer received treatment decision within 30 days after cancer diagnosis. The median waiting time for operation was however more than two months (67 days), and half of the patients received neoadjuvant therapy before the operation. Reduction in the medical delays before therapeutic intervention has proven to be of paramount importance for critical and urgent conditions such as stroke [17], myocardial infarction [18]. However, the importance of medical delays for chronic diseases including cancer is inconclusive. Most studies found null impact of waiting time on cancer specific survival, including gastro-esophageal [7, 8, 16], colorectal [19], lung [20], and pancreatic [6] cancers. Other studies claimed however that a detrimental effect on the prognosis could be introduced if certain waiting time threshold was exceeded. For example, prolonged interval between diagnostic imaging and resection over 32 days for pancreatic cancer was associated with significantly increased risk of unexpected tumor progress as determined at laparotomy [21]. A recent population-based study [22] emphasized a significantly lower overall survival among patients with non-metastatic invasive breast cancer after 30 days waiting time for surgery (approximately 10% decreasing survival for every 30-day increment in waiting). Similarly, shorter overall survival has been suggested for 60 and more days of waiting time for head and neck cancers [23, 24], and for 12 and more days of waiting time for uterine cancers [25].

The psychological consequences of lengthy waiting time for treatment among cancer patients remain largely unknown. Our finding of the inverse assocation between waiting time and new onset mental disorders corroborates is unexpected, but corroborates the findings of a descriptive study including 21 breast cancer patients [26], which hypothesized that a proper interval between cancer diagnosis and start of treatment might be necessary and beneficial in terms of facilitating psychological adaptation [27]. A retrospective Dutch study including 202 breast cancer patients also implied that waiting time for diagnosis, but not waiting time for surgery, could affect the emotional well-being of cancer patients [28]. Considering that the pressure of providing speedy treatment might lead to reduced care quality [29, 30], short waiting time scheme should be designed and utilized with full caution.

Nevertheless, for patients with a history of mental disorders, the necessity of speedy cancer treatment seems to be justified. Clinical studies [31–33] indicated a similar cancer incidence but excess cancer mortality among patients with psychiatric diagnoses, compared to individuals without such comorbidity. Delayed diagnosis, and therefore a presentation at an advanced tumor stage at cancer diagnosis might be one possible explanation underlying such excess mortality [34]. However, UK researchers [35] observed a worse survival outcome for cancer patients with a history of mental illness, although these patients did indeed not have more advanced cancers at diagnosis, suggesting that the differential survival might instead be driven by factors during the process of cancer care after diagnosis. In the present study, we demonstrated that patients with history of psychiatric disorders with longer waiting times for cancer treatment had increased needs of hospital care for mental health during the first year after treatment. Psychiatric symptoms might affect patients’ physical health, responsiveness to cancer treatment, and compliance to treatment regime (e.g. tolerance to intensive regimes) [33, 36], leading to altered overall survival in the long run. Improved prognosis might therefore be expected as a consequence of investment in timely treatment for such patients.

The major strength of our study is the large-scale population-based cohort design, with all information regarding cancer diagnosis, treatment, and mental disorders collected prospectively and independently. The linked data from death and emigration registers ensured the completeness of follow-up information. The availability of detailed questionnaire data from the quality register enabled considerations of a wide range of patient and tumor characteristics during the analysis.

Lack of data on the exact starting date of cancer treatment is a limitation. But the similar results of the sensitivity analysis by restricting to patients with surgical treatment alone relieved this concern. Further, the application of drug register for case identification, on one hand, increased the sensitivity by capturing undiagnosed patients who experienced difficulties in emotional responses [37], while reduced the possibility of underestimation caused by the prioritized cancer management―this might be especially true for patients in the shortest waiting group (i.e., more advanced diseases), being indicated by inconsistent HRs observed for diagnosed mental disorders and disorders identified by medication. However, on the other hand, the misclassification induced by this broad definition might exist, considering that antidepressant drug was not exclusively used for mental problems among cancer patients.

The potential influence of uneven mortality rates between patients with different waiting times (i.e., competing risks) on the present findings needs to be considered. However, informative censoring should have been alleviated sufficiently after adjusting for all prognostic indicators (i.e. cancer stage, therapy type, admission pattern, etc.) in the regression models. Also, similar results from subgroups analysis further relieved such concern. Moreover, although we cannot rule out possible residual confounding due to shared risk factors between cancer and mental disorders, the divergent result patterns between patients with and without mental disease history argues clearly such possibility.

## Conclusions

In conclusion, our study suggests the treatment delays after a diagnosis of esophageal or gastric cancer might be detrimental for the mental health of patients with preexisting mental disorder, at least during the first year after treatment. For patients without a mental disorder history, however, longer waiting times seem to be linked with a lower risk of new onset mental disorders after treatment.

## Additional files

Additional file 1: Table S1. Subgroup analyses for patients with different characteristics. (DOC 74 kb)
Additional file 2: Table S2. Sensitivity analysis, restricted to patients with surgical treatments alone. (DOC 33 kb)

## Abbreviations

ASA: The American Society of Anesthesiologists
CI: Confidence interval
HR: Hazard ratio
ICD: International Classification of Diseases codes
MDC: Multidisciplinary
NREV: National Quality Register for Esophageal and Gastric Cancers
