# Supplementary material for: Waiting time for cancer treatment and mental health among patients with newly diagnosed esophageal or gastric cancer: a nationwide cohort study
Source: BMC Cancer. 2017 Jan 3;17:2. doi: 10.1186/s12885-016-3013-7 (PMC5209901; doi:10.1186/s12885-016-3013-7)
Supplement: Additional file 1: Table S1. — Subgroup analyses for patients with different characteristics. (DOC 74 kb) [file 12885_2016_3013_MOESM1_ESM.doc]

Table S1 Subgroup analyses for patients with different characteristics

| Subgroups | Waiting time groups  Hazard Ratios (HRs) and 95 % Conﬁdence Intervals (CIs)* | | | |
| --- | --- | --- | --- | --- |
|  | 1-8 days | 9-17 days | 18-29 days | 30-60 days |
| *By age group* |  |  |  |  |
| <64 years | 1.11 (0.82-1.48) | 1.00 (reference) | 0.90 (0.68-1.18) | 0.66 (0.48-0.89) |
| 64-71 years | 1.01 (0.71-1.43) | 1.00 (reference) | 0.84 (0.62-1.15) | 0.62 (0.45-0.87) |
| 72-80 years | 1.19 (0.86-1.64) | 1.00 (reference) | 0.69 (0.49-0.98) | 0.67 (0.48-0.92) |
| ≥81 years | 1.34 (0.96-1.86) | 1.00 (reference) | 0.79 (0.55-1.15) | 0.72 (0.50-1.04) |
| By gender |  |  |  |  |
| Men | 1.11 (0.92-1.34) | 1.00 (reference) | 0.75 (0.63-0.91) | 0.61 (0.50-0.74) |
| Women | 1.26 (0.95-1.68) | 1.00 (reference) | 0.92 (0.69-1.22) | 0.78 (0.58-1.04) |
| *By material status* |  |  |  |  |
| Single | 1.31 (0.81-2.10) | 1.00 (reference) | 0.89 (0.55-1.46) | 0.81 (0.49-1.34) |
| Married | 1.15 (0.93-1.43) | 1.00 (reference) | 0.83 (0.67-1.01) | 0.62 (0.50-0.77) |
| Divorce | 0.83 (0.54-1.29) | 1.00 (reference) | 0.54 (0.34-0.87) | 0.67 (0.43-1.03) |
| Widow/widower | 1.47 (1.02-2.12) | 1.00 (reference) | 0.80 (0.54-1.20) | 0.74 (0.50-1.09) |
| *By education level* |  |  |  |  |
| ≤ 9 years | 1.18 (0.94-1.49) | 1.00 (reference) | 0.70 (0.55-0.89) | 0.55 (0.43-0.71) |
| 10-12 years | 1.18 (0.89-1.56) | 1.00 (reference) | 0.80 (0.61-1.06) | 0.77 (0.58-1.01) |
| >=12 years | 1.01 (0.65-1.57) | 1.00 (reference) | 1.17 (0.80-1.69) | 0.88 (0.59-1.31) |
| *By hospital volume* |  |  |  |  |
| low | 1.12 (0.90-1.40) | 1.00 (reference) | 0.73 (0.58-0.93) | 0.62 (0.48-0.79) |
| median | 1.21 (0.87-1.68) | 1.00 (reference) | 0.80 (0.58-1.11) | 0.82 (0.59-1.15) |
| high | 1.23 (0.90-1.69) | 1.00 (reference) | 0.91 (0.69-1.20) | 0.63 (0.47-0.83) |
| *By multidisplinary meeting* | |  |  |  |
| Yes | 1.03(0.80-1.32) | 1.00 (reference) | 0.88 (0.71-1.09) | 0.72 (0.58-0.90) |
| No | 1.04(0.78-1.38) | 1.00 (reference) | 0.77(0.57-1.05) | 0.68(0.49-0.93) |
| *By ASA physical status* |  |  |  |  |
| I-II | 1.17 (0.97-1.42) | 1.00 (reference) | 0.80 (0.67-0.97) | 0.71 (0.58-0.86) |
| III-IV | 1.21 (0.91-1.62) | 1.00 (reference) | 0.87 (0.64-1.18) | 0.55 (0.40-0.75) |
| *By cancer type* |  |  |  |  |
| Gastric cancer | 1.38 (1.11-1.70) | 1.00 (reference) | 0.80 (0.64-1.01) | 0.64 (0.50-0.81) |
| Esophageal cancer | 0.96 (0.76-1.23) | 1.00 (reference) | 0.81 (0.65-1.01) | 0.68 (0.54-0.84) |
| *By stage* |  |  |  |  |
| Stage III-IV | 1.20 (1.00-1.43) | 1.00 (reference) | 0.74 (0.61-0.89) | 0.61 (0.50-0.74) |
| Stage 0-II | 0.97 (0.68-1.36) | 1.00 (reference) | 0.95 (0.71-1.27) | 0.78 (0.58-1.05) |
| *By follow-up period* |  |  |  |  |
| ≤1 year | 1.05 (0.98-1.25) | 1.00 (reference) | 0.84 (0.70-1.00) | 0.76 (0.63-0.92) |
| >1 year | 1.19 (0.79-1.78) | 1.00 (reference) | 1.01 (0.70-1.48) | 0.92 (0.63-1.39) |

*Adjusted for age, sex, marital status (single, married, divorce, widow/widower), education level (<9 years, 9-12 years, >12 years), physical status (The American Society of Anesthesiologists (ASA) classification <2, 2 and above), cancer type (esophageal/gastric cancer), stage (0, I, II, III, IV stage), and planned treatment type (curative, palliative, or supportive treatment), multidisplinary meeting (yes/no), admission pathway (general physician referral / emergency intake), hospital volume (low, median, high), with the exception that this factor was used for subgrouping.
