# Supplementary material for: Waiting time for cancer treatment and mental health among patients with newly diagnosed esophageal or gastric cancer: a nationwide cohort study
Source: BMC Cancer. 2017 Jan 3;17:2. doi: 10.1186/s12885-016-3013-7 (PMC5209901; doi:10.1186/s12885-016-3013-7)
Supplement: Additional file 2: Table S2. — Sensitivity analysis, restricted to patients with surgical treatments alone. (DOC 33 kb) [file 12885_2016_3013_MOESM2_ESM.doc]

Table S2 Sensitivity analysis, restricted to patients with surgical treatments alone

| Outcomes | Waiting time groups* | | | |
| --- | --- | --- | --- | --- |
|  | 1-26 days | 27-41 days | 42-59 days | ≥60 days |
| *Among patients without mental disorder history (n=918)* | | | | |
| All mental disorders | 1.28 (0.90-1.76) | 1.00 (reference) | 1.04 (0.72-1.50) | 0.94 (0.65-1.34) |
| *Among patients with mental disorder history (n=437)* | |  |  |  |
| Psychiatric cares within 1 year | 0.70 (0.43-1.14) | 1.00 (reference) | 0.90 (0.62-1.32) | 1.12 (0.77-1.64) |
| Psychiatric cares more than 1 year | 0.59 (0.31-1.09) | 1.00 (reference) | 1.02 (0.61-1.70) | 1.41 (0.85-2.34) |

*Waiting time groups were determined by quartiles of actual waiting days to surgery
